# Supplementary material for: Influenza A virus modulation of Streptococcus pneumoniae infection using ex vivo transcriptomics in a human primary lung epithelial cell model reveals differential host glycoconjugate uptake and metabolism
Source: bioRxiv. 2023 Jan 30:2023.01.29.526157. Preprint. [Version 1] doi: 10.1101/2023.01.29.526157 (PMC9915477; doi:10.1101/2023.01.29.526157)

bioRxiv preprint doi: https://doi.org/10.1101/2023.01.29.526157; this version posted January 30, 2023. The copyright holder for this preprint (which was not certified by peer review) is the author/funder, who has granted bioRxiv a license to display the preprint in perpetuity. It is made available under aCC-BY 4.0 International license.

Supplemental Figure Legends

Supplemental Figure 1. Rarefaction curves of RNA-seq reads mapped to the EF3030 genome and EF3030 colony forming units (CFU). A) Rarefaction curves of EF3030 samples. Samples whose curves plateau indicate sufficient sequencing depth for the majority of EF3030 genes analyzed. B) EF3030 CFU counts for EF3030 monoinfection (no-influenza control, NIC) vs. influenza A virus + EF3030 coinfected nHBEC cells (IAV Spn).

Supplemental Figure 2. Z-scored Heatmaps of differentially expressed (DE) EF3030 gene intersects and principal component analyses (PCA) of EF3030 WGCNA modules in Figure 3A. A) Heatmap of 163 DE genes shared in comparisons of Host+EF3030+pH1N1 vs. EF3030 and Host+EF3030 vs. EF3030, showing genes influenced by nHBEC. B) Heatmap of 80 DE genes shared in all comparisons, showing genes having unique condition-specific levels of expression. C) Heatmap of 52 DE genes shared in comparisons of Host+EF3030+pH1N1 vs. EF3030 and Host+EF3030+pH1N1 vs Host+EF3030, showing genes influenced by pH1N1. Entire DE gene lists are shown in Supplemental Table 3. D-G) PCA of each of the EF3030 WGCNA modules in Figure 3A, black, blue, green, and pink, respectively. Sample clustering and high proportions of variation seen in PC1 (78-88%) suggest the genes in these modules effectively capture condition-specific expression.

Supplemental Figure 3. Z-scored Heatmaps of Regprecise EF3030 regulons having at least 2 genes identified as differentially expressed. Shown are the 9 regulons identified as differentially regulated which had no consistent condition-specific gene expression, and therefore were not shown in Figure 4.

Supplemental Figure 4. Spearman correlations of RNA-Seq vs. Nanostring for 63 EF3030 genes. X axes are DESeq2 Variance Stabilized Transformation (VST) counts. Y axes are normalized Nanostring expression values. Both axes are on a Log_2 scale.

Supplemental Figure 5. TIGR4 wildtype vs. TIGR4 knockout GlnR regulon mutant growth curves. Optical density measurements of *Streptococcus pneumoniae* TIGR4 wt/mutant growth over 6-7 hours in liquid media.

Supplemental Figure 1.

A

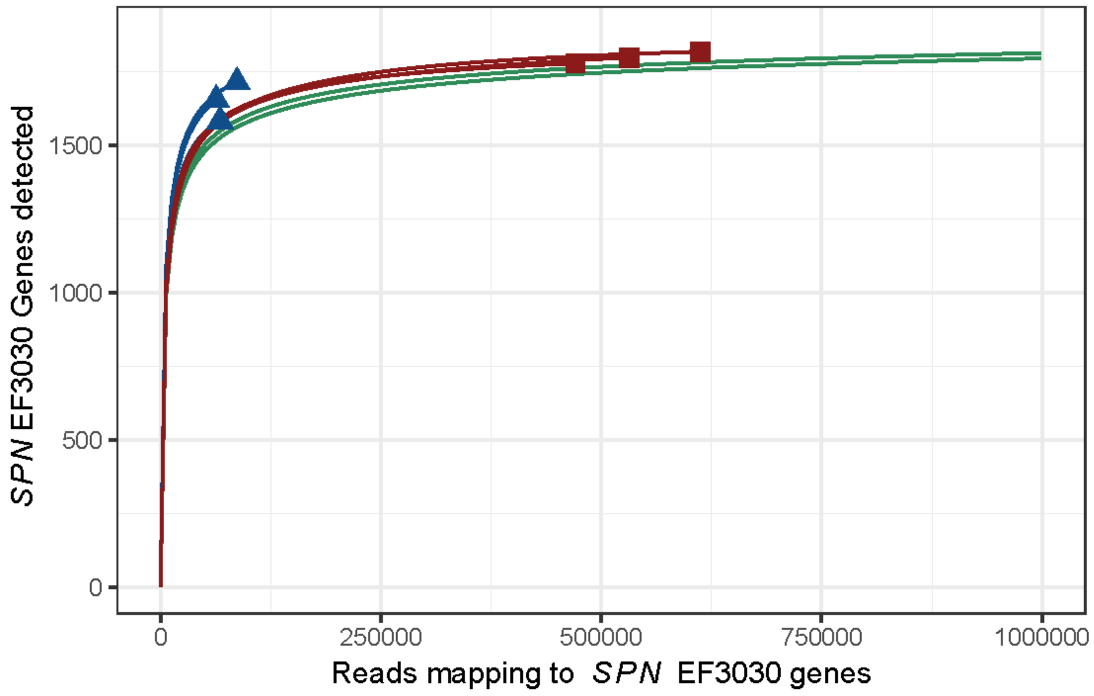

B

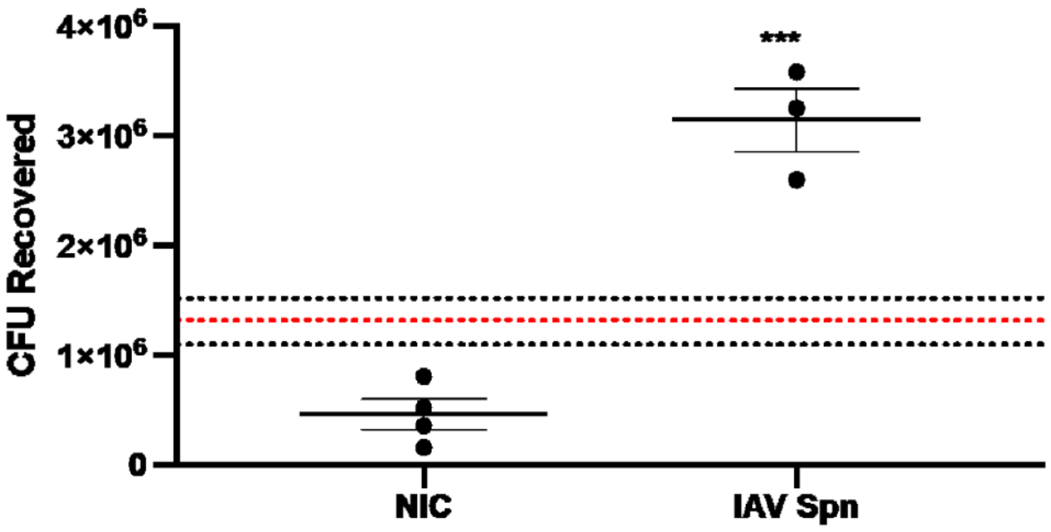

(which was not certified by peer review) is the author/funder, who has granted bioRxiv a license to display the preprint in perpetuity. It is m
available under aCC-BY 4.0 International license.

Supplemental Figure 2.

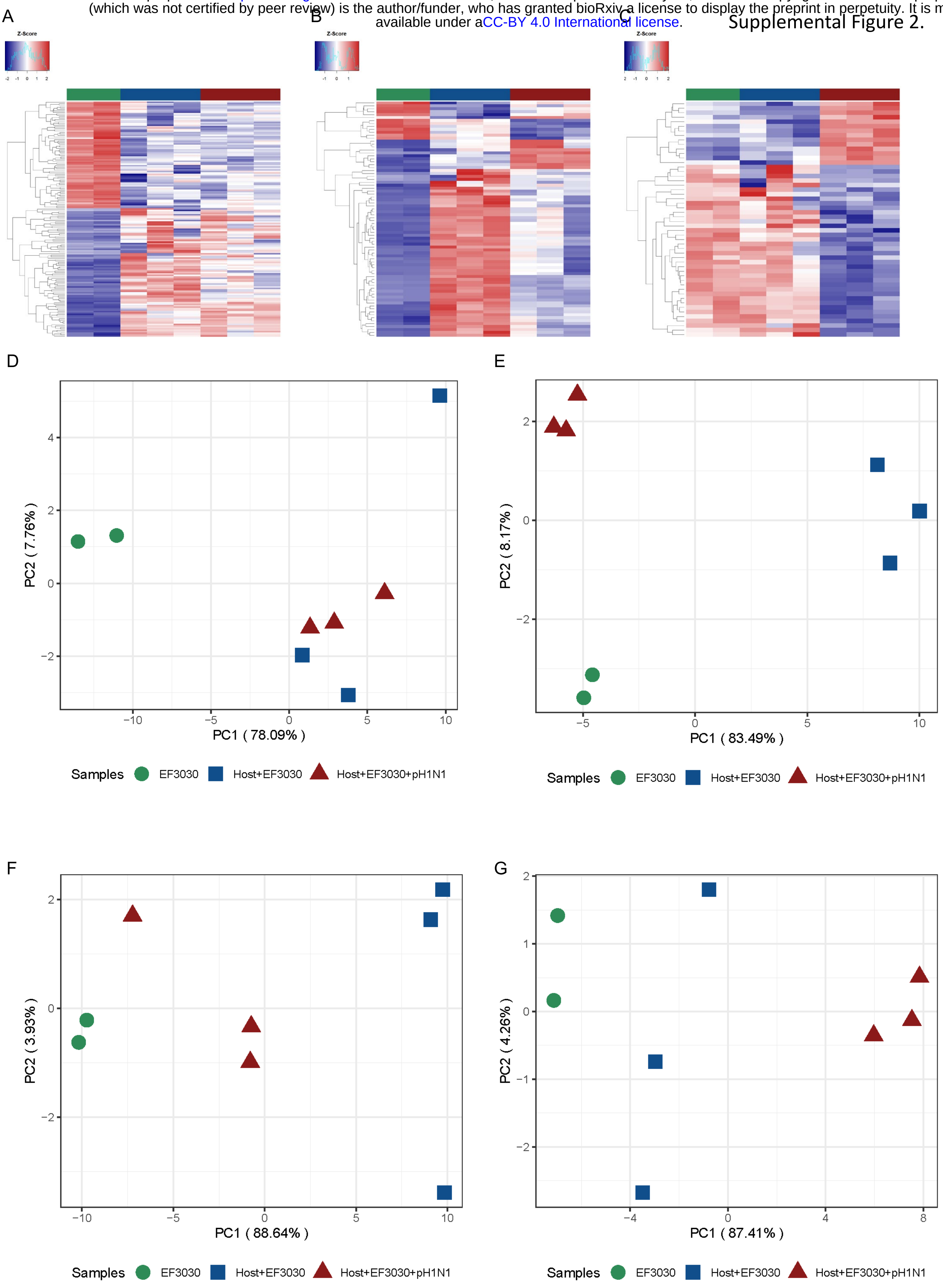

(which was not certified by peer review) is the author/funder, who has granted bioRxiv a license to display the preprint in perpetuity. It is m
available under aCC-BY 4.0 International license.

Supplemental Figure 3.

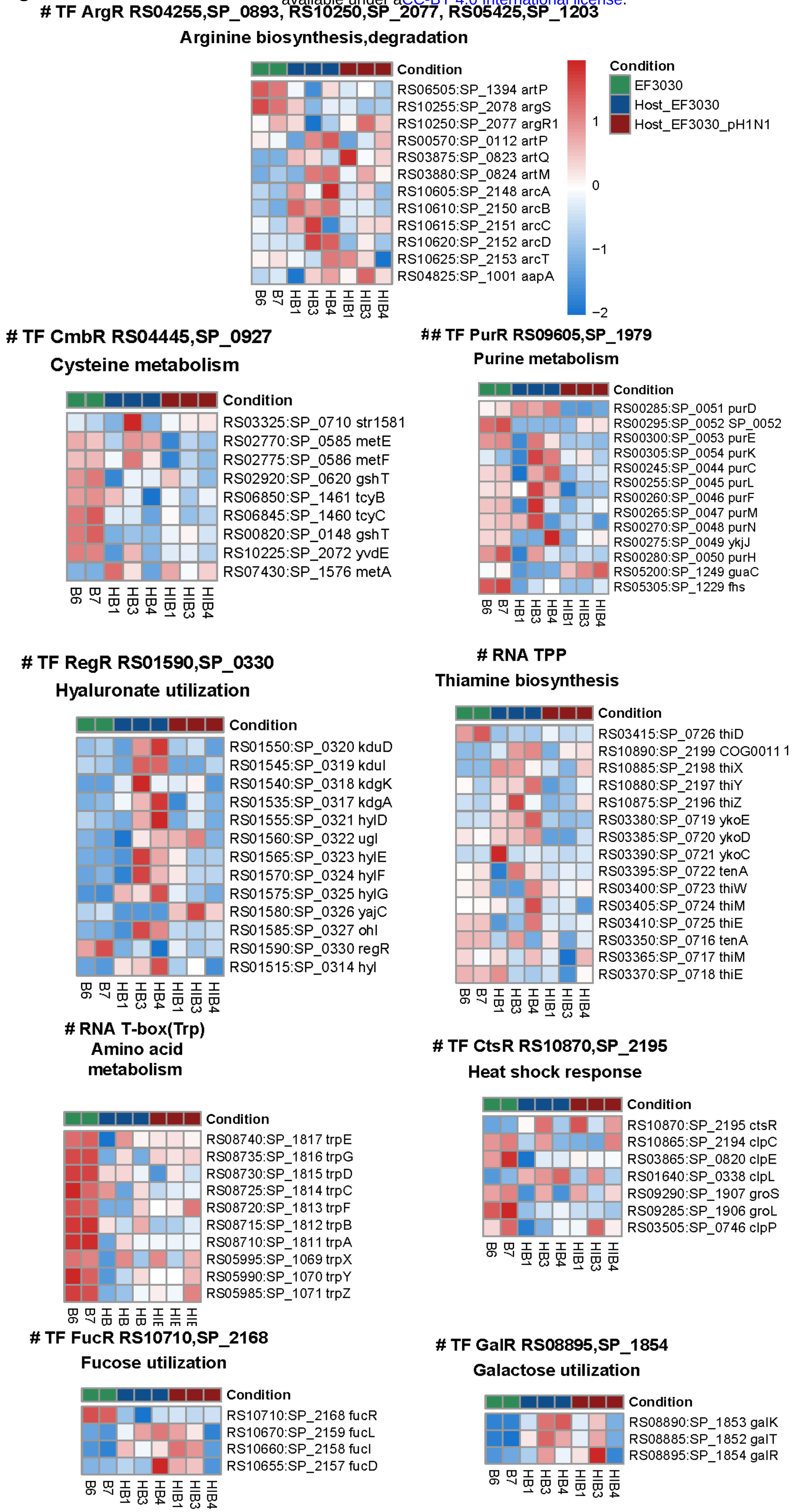

(which was not certified by peer review) is the author/funder, who has granted bioRxiv a license to display the preprint in perpetuity. It is m
available under aCC-BY 4.0 International license.

Supplemental Figure 4.

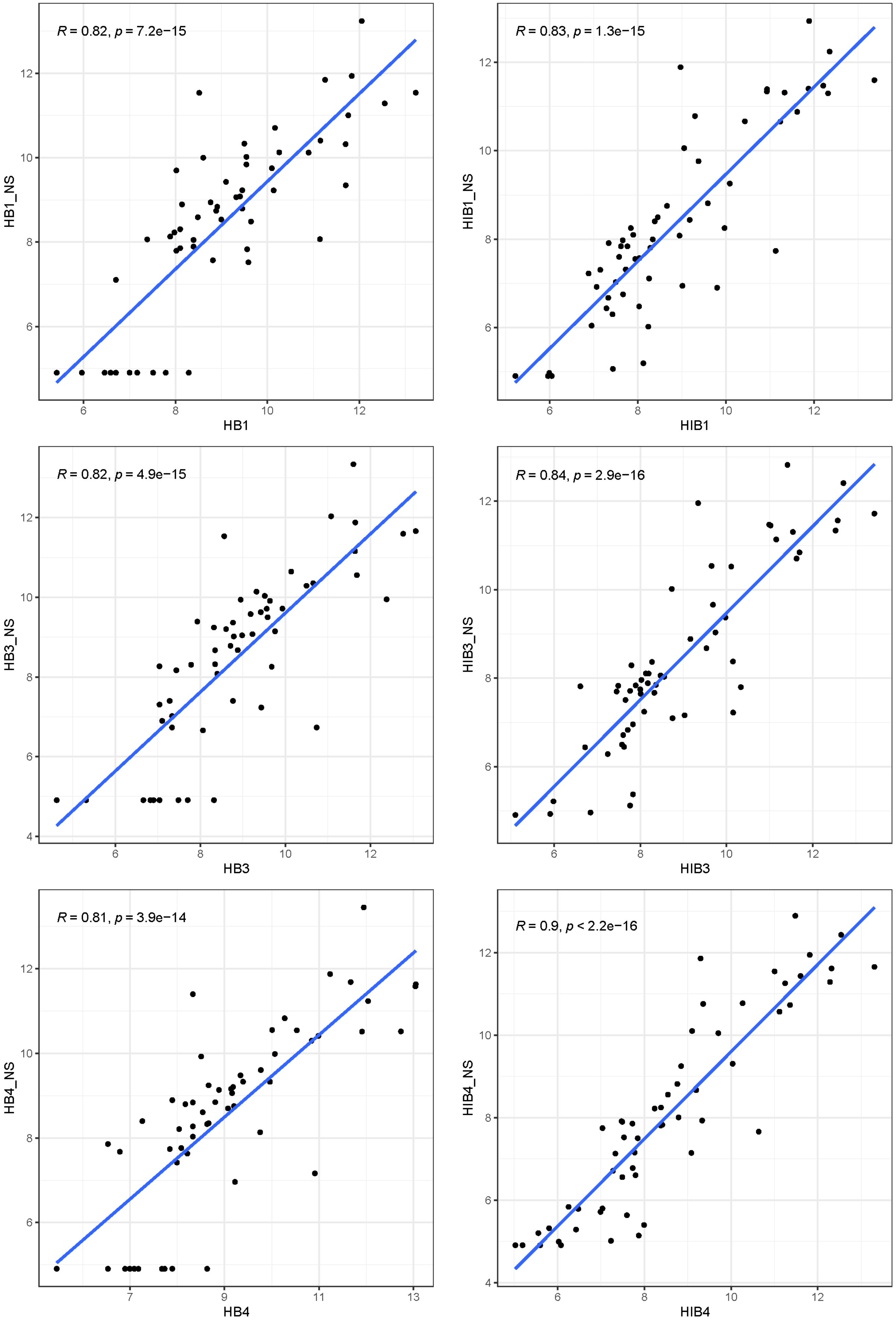

Supplemental Figure 5.

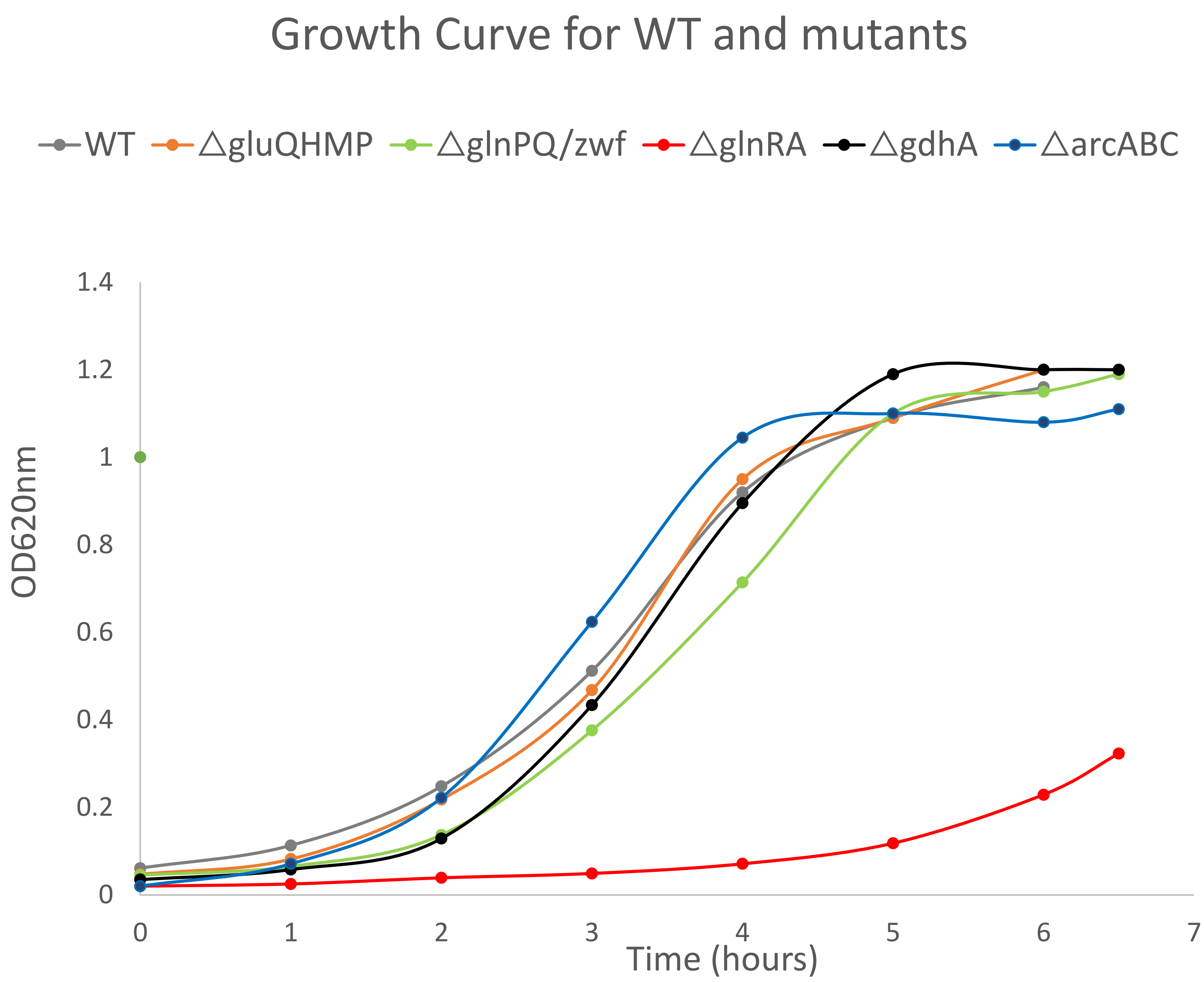

Supplement: Supplement 1 [file NIHPP2023.01.29.526157v1-supplement-1.pdf]
